# Zika is not a reason for missing the Olympic Games in Rio de Janeiro: response to the open letter of Dr Attaran and colleagues to Dr Margaret Chan, Director - General, WHO, on the Zika threat to the Olympic and Paralympic Games

**DOI:** 10.1590/0074-02760160003

**Published:** 2016-06

**Authors:** Claudia Codeço, Daniel Villela, Marcelo F Gomes, Leonardo Bastos, Oswaldo Cruz, Claudio Struchiner, Luis Max Carvalho, Flavio Coelho

**Affiliations:** 1 Fundação Oswaldo Cruz, Programa de Computação Científica, Rio de Janeiro, RJ, Brasil; 2 Fundação Getúlio Vargas, Rio de Janeiro, RJ, Brasil

**Keywords:** Zika, Olympic Games

## Abstract

Attaran and colleagues in an open letter to WHO expressed their concern about the upcoming Olympic and Paralympic Games in Rio de Janeiro and the threat posed by the Zika epidemic (Attaran 2016). We agree that Zika virus is of great public health concern and much remains to be known about this disease. Care should be taken to reduce the risk of infection, especially to pregnant women. However, we argue that this is not sufficient reason for changing the original plans for the Games, in particular because of the time of the year when they will take place. The present article outlines several scientific results related to Zika and mosquito-borne infectious diseases dynamics that we believe ratify the current position of WHO in not endorsing the postponing or relocation of the 2016 Olympic and Paralympic Games (WHO 2016).

*Risk of Zika infection during the Olympic Games* - August is winter in Rio de Janeiro, cool and dry, with daily temperatures varying between 19ºC-26ºC. Although *Aedes aegypti* mosquitoes are present year-round, their vectorial transmission capacity is strongly reduced when minimum temperature is below 22-24ºC (Watts et al. 1987). Under these conditions, the extrinsic incubation period, i. e., the time taken for the mosquito to begin transmitting the virus extends to more than two weeks which is more than the average lifespan of mosquitoes in nature (Chan & Johansson 2012). Because of this low vectorial capacity, vector borne diseases are at minimum risk during the winter. Figure 1 shows the incidence of dengue fever in Rio de Janeiro, a viral disease also transmitted by *Ae. aegypti*. August and September shows clearly very low activity, with an incidence of one to seven cases for every 100,000 individuals. Previous analyses have shown that the reproduction number of dengue in Rio de Janeiro is only above one (sustained transmission) if minimum temperature > 22ºC (Codeço et al. 2016). Currently, there is no evidence to believe the same should not occur for Zika, considering the mediation by the same mosquito vector. Minimum temperature > 22ºC is only expected by mid-November.

**Figure:**
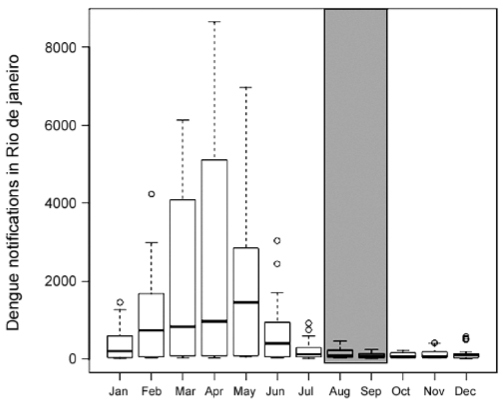
Seasonality of dengue in Rio de Janeiro (data from 2010-2015). The box indicates the date of Olympic and Paralympic Games.

The expected number of tourists coming to Rio de Janeiro is 350,000 to 500,000. Multiplying this by the force of infection of dengue, one obtains an expectation of 4 (1-36) symptomatic dengue cases. This is assuming that tourists would be as exposed as the residents, which is not true since they will be more protected by personal action and not moving around the whole city. The northern part of the city (APS3.3) where dengue is historically more intense (and Zika was as well) is not the epicenter of the game activities (Bastos et al. 2016).

In 2014, similar concern was raised regarding the risk of dengue during the World Cup. Using a mathematical model, Massad et al. (2014)estimated three to 59 symptomatic dengue cases. Ultimately, only three cases of dengue infected tourists were reported during the World Cup (Gautret & Simon 2015). For the Olympic Games, Ximenes et al. (2016) estimated at a worst case scenario 23 to 206 dengue cases among tourists. Such estimate is based on the historical dengue reports from 2007, a year that presented a very low incidence during summer, accompanied by an unusual peak during winter. The present year, however, have already shown a peak of Zika cases in Rio during the summer.

Entomological studies suggest that *Ae. aegypti* from Rio de Janeiro is less efficient at transmitting Zika virus than dengue viruses (Chouin-Carneiro et al. 2016). Thus, the previous numbers can be considered as overestimates. On the other hand, indication of sexual transmission is increasing (Coelho et al. 2016, Mansuy et al. 2016) and preventive measures should be taken, as recommended by WHO. Although recent studies have shown that the reproductive number of Zika outbreak is higher than that of dengue in Rio de Janeiro (Bastos et al. 2016), these analyses correspond to its evolution during local summertime.

In their argument, Dr Attaran (2016) presented figures (incidence, case counts) that correspond to totals, not considering the strong seasonality of *Ae. aegypti* borne diseases. In the notification of Zika in the city, we already observe a decay in case notification since April 2016. They also argue that dengue *“has increased 320% to 1150% over the same periods in 2015 and 2014, respectively”*, however, 2014 had the historically lowest dengue season because of the extreme drought. Actually, in comparison to 2011, 2012 and 2013, the 2015 dengue season had a third of the cases.

*Global Health threat: risk of spreading Zika worldwide* - Dr Attaran and colleagues were also concerned *“about the risk posed when 500,000 foreign tourists from all countries attend the Games, potentially acquire that strain, and return home to places where it can become endemic.”* We argue that the spread most likely already occurred during the Carnival 2016, when Zika activity in Rio de Janeiro was at its peak. During the Carnival festivities, in February 2016, more than 1 million tourists visited Rio de Janeiro, which is twice the number expected for the Olympic Games. Not only Zika case report was at its peak, most Carnival activities took place outdoors, increasing the exposure of tourists to mosquitoes. At the moment, local transmission of Zika virus has already been ascertained in 60 countries (WHO 2016).

It is our belief that the best course of action is not to postpone the Games or to encourage foreigners not to attend, but to inform the population regarding protective measures at the individual level. The best action is a set of practices such as the use of mosquito repellents to avoid bites, which is still assumed to be the main infection route, and the use of condoms to avoid the possibility of sexual transmission, for which evidence is increasing (Coelho et al. 2016, Mansuy et al. 2016), along with public action by Brazilian authorities to minimize exposure to *Ae. aegypti*, which is likely to be facilitated by climate factors during Rio de Janeiro’s winter time.

Postponing the Games to a later time, closer to the summer, is likely to have the inverse effect, based on current knowledge of arboviruses transmission in Rio de Janeiro.

Pregnant women, on the other hand, should avoid travelling to countries with known Zika trasmission, since the severity of the possible outcome in case of an unlikely infection is overwhelming. Nonetheless, the recommendation for the general public is to attend normally, while paying attention to the instructions from WHO and the Brazilian Health authorities.
